# A novel model of non-alcoholic steatohepatitis with fibrosis and carcinogenesis in connexin 32 dominant-negative transgenic rats

**DOI:** 10.1007/s00204-020-02873-5

**Published:** 2020-08-24

**Authors:** Aya Naiki-Ito, Hiroyuki Kato, Taku Naiki, Ranchana Yeewa, Yoshinaga Aoyama, Yuko Nagayasu, Shugo Suzuki, Shingo Inaguma, Satoru Takahashi

**Affiliations:** 1 Department of Experimental Pathology and Tumor Biology, Nagoya City University Graduate School of Medical Sciences, 1-Kawasumi, Mizuho-cho, Mizuho-ku, Nagoya, 467-8601 Japan; 2 Department of Molecular Pathology, Osaka City University Graduate School of Medicine, Osaka, Japan

**Keywords:** NASH, Connexin, Insulin resistance, Fibrosis, Hepatocarcinogenesis

## Abstract

Non-alcoholic steatohepatitis (NASH) is a recognized risk factor for liver fibrosis and malignancies, and is associated with features of metabolic syndrome, such as obesity and insulin resistance (IR). We previously demonstrated that the disturbance of connexin 32 (Cx32), a gap junctional protein of hepatocytes, exacerbated NASH in Cx32 dominant-negative transgenic (Cx32ΔTg) rats fed methionine choline-deficient diet (MCDD). MCDD is well-established means of inducing NASH in rodents; however, the Cx32ΔTg-MCDD NASH model does not reproduce obesity and IR. In this study, we aimed to establish an improved NASH model. Eight-week-old male Cx32ΔTg and wild-type (Wt) rats received a high-fat diet (HFD) with dimethylnitrosamine (DMN) for 12 weeks. The HFD with DMN led to gains in body, liver, and visceral fat weights in both genotypes. IR was significantly greater in Cx32ΔTg than in Wt rats. Elevation of serum hepatic enzymes (AST, ALT), inflammatory cytokine expressions (*Tnfα*, *Il-6*, *Tgf-β1*, *Il-1β*, *Timp2*, and *Col1a1*), steatohepatitis, and fibrosis were significantly greater in Cx32ΔTg as compared with Wt rats. Regarding carcinogenesis, the number and area of glutathione S-transferase placental form (GST-P)-positive preneoplastic hepatic foci were significantly increased in Cx32ΔTg versus Wt rats. Moreover, activation of NF-κB and JNK contributed to the progression of NASH in Cx32ΔTg rats. These results suggest that Cx32 dysfunction promoted the progression of NASH, metabolic syndrome, and carcinogenesis. Therefore, the novel Cx32ΔTg–HFD–DMN NASH model may be a rapid and useful tool for evaluating the progression of NASH.

**Electronic supplementary material:**

The online version of this article (10.1007/s00204-020-02873-5) contains supplementary material, which is available to authorized users.

## Introduction

Non-alcoholic fatty liver disease (NAFLD) is recognized as a hepatic manifestation of metabolic syndrome related to obesity, insulin resistance (IR), and glucose intolerance (Chalasani et al. 2012). NAFLD is defined as the presence of steatosis in the absence of other cause of chronic liver disease, such as viral infection or alcohol consumption. A global meta-analytic study using the MEDLINE/PubMed database indicated that the overall global prevalence of NAFLD was 25.24%, with the highest prevalence in South America and the Middle East and the lowest in Africa (Younossi et al. 2016). Non-alcoholic steatohepatitis (NASH) is a more aggressive form of liver disease defined as steatosis with histological hepatocyte injury (Anstee et al. 2013; Chalasani et al. 2012), and it has been detected in 59.1% of biopsied NAFLD patients (Younossi et al. 2016). Similar to other chronic liver diseases, such as viral hepatitis and alcohol-induced damage, NASH has the potential to lead to the development of cirrhosis and hepatocellular carcinoma (HCC) and, therefore, represents a serious public health problem (Bugianesi et al. 2002). The rise in the incidence of HCC in patients with NASH has been attributed to an increase in the number of cases of NASH with cirrhosis; however, recent studies demonstrate that HCC may also arise in NAFLD or NASH in the absence of cirrhosis (Torres and Harrison 2012).

Connexins (Cxs) are subunits of gap junction channels, which allow the exchange of small molecules (< 1 kDa), such as ions, second messengers, and cellular metabolites between adjacent cells (Evans and Martin 2002; Loewenstein 1981), and plays an important role in tissue homeostasis and the control of cell growth and differentiation (Trosko and Chang 2001; Yamasaki 1990). Cx32 and Cx26 are major gap junctional proteins of hepatocytes; Cx32 is expressed throughout hepatocyte lobules, and Cx26 is localized at the periportal zone (Paul 1986; Sagawa et al. 2015). Cx32 is involved in hepatocarcinogenesis in both rodents and human. The expression of Cx32 gradually decreases during the progression of chronic liver diseases including viral hepatitis, cirrhosis, and HCC in humans (Nakashima et al. 2004). Decreased Cx32 expression associated with aging has been shown to correlate with an increase in hepatocarcinogenesis (Naiki-Ito et al. 2012). We previously established a line of transgenic rats (Cx32ΔTg) carrying a dominant-negative mutation of Cx32, under the control of an albumin promoter, allowing us to examine hepatocyte-specific Cx32 function (Asamoto et al. 2004). Cx32ΔTg rats showed diffusely disturbed hepatic membrane expression of endogenous Cx32/Cx26 proteins and greatly decreased gap junctional intercellular communication. Even though no toxic effect was observed in untreated Cx32ΔTg rats, disruption of Cx32 remarkably reduced chemically induced hepatotoxicity of carbon tetrachloride and acetaminophen through inhibition of apoptosis in Cx32ΔTg rats (Asamoto et al. 2004; Naiki-Ito et al. 2012). Conversely, Cx32ΔTg rats exhibit higher carcinogenic susceptibility as compared with wild-type (Wt) littermates (Hokaiwado et al. 2005, 2007). These findings indicate that Cx32 may play a crucial role in the metabolism of chemicals and maintenance of homeostasis in the liver.

Several methods can be used for the experimental induction of NASH. A high-fat diet (HFD) can be used to induce fatty liver in rodents; however, this model does not consistently reproduce inflammation and hepatocyte injury. The most widely used model of NASH involves the feeding of chorine- and/or methionine-deficient diet, which induces not only steatosis but also active infiltration of inflammatory cells and hepatocyte injury (Tanaka et al. 2012). However, the ingestion of a methionine choline-deficient diet (MCDD) does not induce fibrosis in rodents rapidly (Yamaguchi et al. 2008). Recently, we reported that MCDD treatment induces more severe steatohepatitis and fibrosis in Cx32ΔTg as compared with Wt rats. In accordance with the results in the Cx32ΔTg-MCDD model, dysfunction of Cx32 accelerates NASH and fibrosis progression via an increase in reactive oxygen species (ROS), inflammatory cytokines, and activation of the brain expressed, X-linked 1 (Bex1)-NF-κB pathway (Sagawa et al. 2015). Therefore, Cx32ΔTg rats may be a suitable model for evaluating histological changes in NASH. However, obesity and IR, which reflect metabolic syndrome, do not occur in models in which NASH is induced by MCDD.

In this study, we examined a new experimental protocol to induce NASH using Cx32ΔTg rats, which involved the feeding of an HFD to trigger steatosis. In addition, we administered dimethylnitrosamine (DMN), which is a liver carcinogen and has the potential to drive hepatic fibrosis (Matsuda et al. 1997; Pan et al. 2015), combined with the HFD.

## Materials and methods

### Production and screening of transgenic rats

The establishment, production, and screening of Cx32ΔTg rats were carried out as previously described in detail (Asamoto et al. 2004; Naiki-Ito et al. 2010). Rats were maintained in plastic cages on hardwood chips, in an air-conditioned, specific pathogen-free animal room at 22 ± 2 °C and 50% humidity with a 12 h/12 h light–dark cycle. Animals were given free access to tap water. All animal experiments were performed under protocols approved by the Institutional Animal Care and Use Committee of Nagoya City University School of Medical Sciences (no. H27M-65, approved on November 24, 2015).

### Animal treatments

**Fig. 1:**
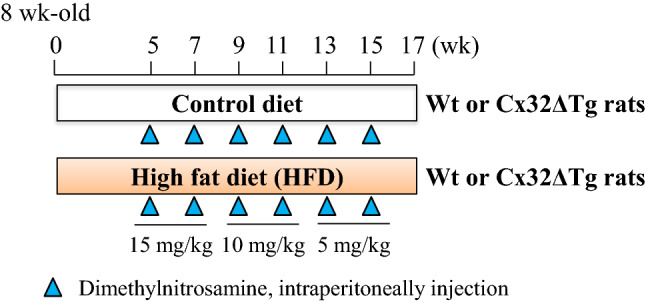
Experimental schedules for the development of steatohepatitis and fibrosis. Male Cx32 dominant-negative transgenic (Cx32ΔTg) and wild-type (Wt) rats at 8 weeks of age received control diet (Ctrl) or high-fat diet (HFD) for 17 weeks. At week 5, dimethylnitrosamine (DMN) was intraperitoneally administered 6 times in total with 1 injection every 2 weeks. The dosage of DMN was 15 mg/kg (first and second injections), 10 mg/kg (third and fourth injections), and 5 mg/kg (fifth and sixth injections)

The experimental design is illustrated in Fig. 1. Male Cx32ΔTg rats and Wt littermates at 8 weeks of age received a control diet (AIN-93 M; Oriental BioService, Inc., Kyoto, Japan) or an HFD (HFD-60, Oriental BioService, Inc.) for 17 weeks. At 5 weeks, DMN (Tokyo Kasei Kogyo Co. Ltd, Tokyo, Japan) was injected intraperitoneally 6 times with injection administered once every 2 weeks. The ideal dosage of DMN was determined to be 15 mg/kg (first and second injections), 10 mg/kg (third and fourth injections), and 5 mg/kg (fifth and sixth injections) based on the dosage (10 mg/kg a day) used in a previous DMN-induced rat liver fibrosis model (Matsuda et al. 1997). The dosage during the experiment was gradually reduced according to data of the body weight gain per week. The four groups studied were Wt-Control (Ctrl) (*n* = 16), Wt-HFD (*n* = 21), Cx32ΔTg-Ctrl (*n* = 16), and Cx32ΔTg-HFD (*n* = 21). Six rats in each group were sacrificed at the week 5 before treatment with DMN began, and other rats were sacrificed at the week 17 following the feeding of the experimental diets. The livers and fat surrounding the epididymis were immediately excised, weighed, and cut into slices 3-to-4 mm thick. They were then fixed in 10% buffered formalin, embedded in paraffin, and routinely processed for histological evaluation (2–3 μm thick). Additional liver tissue was frozen and stored at − 80 °C until processing. The frozen samples were used for the extraction of protein and total RNA.

### Biochemical analysis of blood samples

Blood was collected by puncture of the abdominal aorta at the sacrifice. Plasma total protein (TP), albumin (Alb), total cholesterol (T-chol), low-density lipoprotein cholesterol (LDL), high-density lipoprotein cholesterol (HDL), aspartate aminotransferase (AST), alanine aminotransferase (ALT), and alkaline phosphatase (ALP) were measured using standard procedures on an autoanalyzer by a commercial laboratory [The Tohkai Cytopathology Institute: Cancer Research and Prevention (TCI-CaRP), Gifu, Japan]. Insulin was analyzed using a Morinaga Ultra Sensitive Rat Insulin ELISA Kit (Morinaga Institute of Biological Science, Yokohama, Japan). Homeostasis model assessment-insulin resistance (HOMA-IR) scores were calculated to determine IR (HOMA-IR = fasting insulin (µU/ml)/fasting glucose (ml/dl)/405) (Matthews et al. 1985).

### Histological analysis of NASH

The degree of steatohepatitis was evaluated as previously described in detail (Sagawa et al. 2015). Briefly, formalin-fixed liver sections were stained with hematoxylin and eosin (H&E) or Azan, and were also used for immunohistochemical measurement of α-smooth muscle actin (α-SMA, Dako, Tokyo, Japan). The positive areas of Azan and α-SMA immunostaining were measured with an image analyzer (Keyence, Osaka, Japan). The progression of steatohepatitis was analyzed using a non-alcoholic fatty liver disease activity score (NAS): the score represents a sum of 3 subscores; namely, severity of steatosis (0–3), lobular inflammation (0–2), and hepatocyte ballooning (0–3). NAS and scores for fibrosis (0–4) were determined by three experienced pathologists (AN, HK, and ST), according to the method described by Kleiner et al. (2005).

### Identification of preneoplastic foci in the liver

Immunohistochemical staining of glutathione *S*-transferase placental form (GST-P) was performed in accordance with our previous report (Naiki-Ito et al. 2012). The average number and area of GST-P-positive foci > 80 μm in diameter in the total area of the liver section were measured with an image analyzer (Keyence, Osaka, Japan).

### Western blotting

Frozen liver tissues collected at week 17 were homogenized with RIPA buffer (Thermo Fisher Scientific, Rockford, IL) containing protease and phosphatase inhibitors (Thermo Fisher Scientific). Protein concentrations were quantified by the Bradford procedure and equal amounts of proteins were used as samples. Samples (30 μg per lane) were loaded and separated on 12% acrylamide gels and electroblotted onto nitrocellulose membranes (Hybond-ECL, GE Healthcare UK Ltd., Buckinghamshire, UK). The primary antibodies used were against the following antigens: nuclear factor-κB (NF-κB), phosphorylated (p)NF-κB (Ser536), IκB-α, Cdc42, Mkk4, pMkk4 (Ser80 and Thr261), Jnk, pJnk (Thr183/Tyr185), pc-Jun (Ser63) (Cell Signaling Technology, Danvers, MA), Cx32 (Thermo Fisher Scientific), Cx26 (Thermo Fisher Scientific), and β-actin (Sigma-Aldrich, St. Louis MO). The dilution ratios were 1:5000 for anti-β-actin, 1:500 for anti-Cx32 and anti-Cx26, and 1:1000 for all other antibodies. The intensity of each band was measured using Image J software, ver. 1.46 (National Cancer Institute Bethesda, MD).

### RNA extraction and quantitative reverse transcription PCR

Total RNA was isolated from frozen liver tissues collected at week 17 by phenol–chloroform extraction (Isogen, Nippon Gene Co. Ltd., Tokyo, Japan). One microgram of RNA was converted to cDNA with Moloney Murine Leukemia Virus reverse transcriptase (Takara, Otsu, Japan) in a 20 μL reaction mixture. Aliquots (2 μL) of cDNA samples were subjected to quantitative PCR in a total volume of 25 μL using SYBR Premix ExTaq II (Takara) in a light cycler apparatus (Roche Diagnostic Basel, Switzerland). The primers used are listed in Table S1. Gapdh mRNA levels were used as internal controls.

### Statistical analysis

Differences in quantitative data, expressed as mean ± standard deviation (SD), between groups were compared by one-way ANOVA with Tukey multiple comparison tests using Graph Pad Prism 8 (GraphPad Software, Inc., La Jolla, CA). A *P* value < 0.05 was considered significant.

## Results

### HFD induces steatohepatitis in Cx32ΔTg rats

We initially investigated the development of NASH following 5 weeks of HFD diet consumption in Wt and Cx32ΔTg rats. The body, liver, and fat weights were significantly higher in HFD groups as compared with control groups in both Wt and Cx32ΔTg rats (Table S2). H&E staining revealed that parenchymal fat deposition containing tiny or large lipid droplets was induced throughout the lobule of the liver in the HFD groups (Fig. S1a and S1b); there was no significant difference between the two genotypes. Neutrophil infiltration was significantly increased in Cx32ΔTg as compared with Wt rats (Fig. S1a and S1c; *P* < 0.05), and hepatocyte ballooning was evident in only two rats in the Cx32ΔTg-HFD group. Consequently, NAS was significantly elevated in the HFD groups as compared with the control groups in both genotypes and showed a trend to be higher in the Cx32ΔTg-HFD than in the Wt-HFD group (Fig. S1d; *P* = 0.056). Reflecting histological changes in the liver, the serum level of ALT was significantly raised by HFD feeding in both genotypes (Table S3). These results indicate that simple HFD supplementation induced more severe steatohepatitis in Cx32ΔTg than in Wt rats.

### Combination of HFD with DMN induce steatohepatitis and insulin resistance in Cx32ΔTg rats

**Fig. 2:**
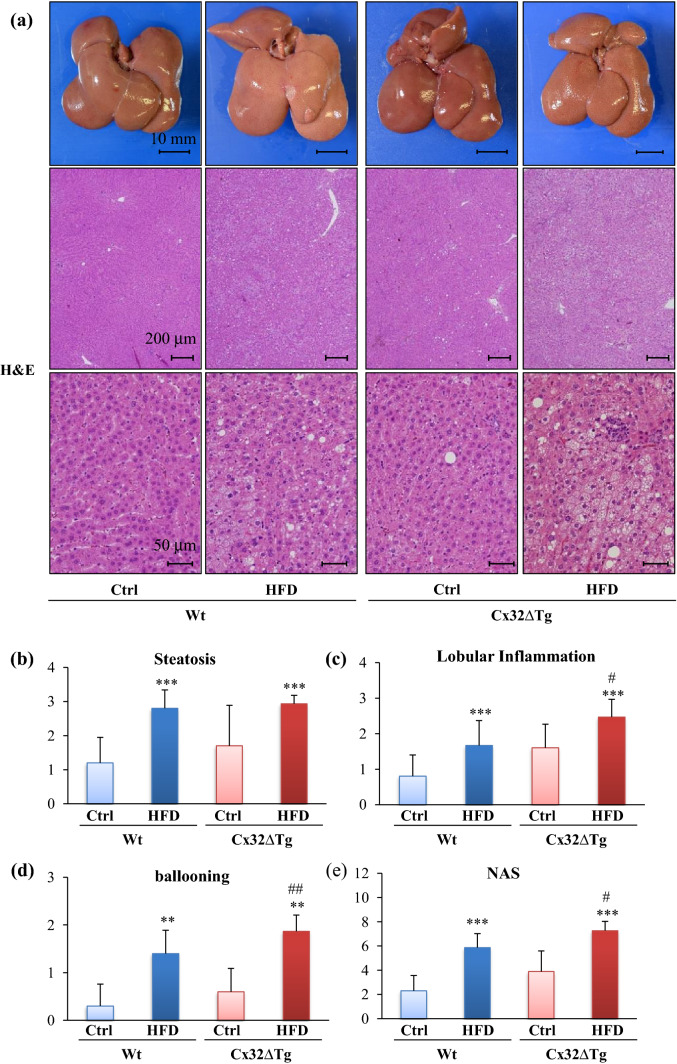
Steatohepatitis induced by high-fat diet (HFD) and dimethylnitrosamine (DMN) in Cx32 dominant-negative transgenic (Cx32ΔTg) rats. **a** Representative macroscopic images and hematoxylin and eosin (H&E) staining of liver sections from the Control (Ctrl), HFD-treated wild-type (Wt), and Cx32ΔTg groups at week 17. Histopathological analysis of steatohepatitis with severity scores for steatosis (**b**), lobular inflammation (**c**), hepatocellular ballooning (**d**), and non-alcoholic fatty liver disease activity score (NAS) (**e**). Data are presented as mean ± SD, *n* = 16–21 per group, **P <0.01, ***P <0.001 statistically significant between genotype-matched Ctrl and HFD groups, ^#^*P *<0.05, ^##^*P *<0.01 statistically significant between treatment-matched Wt and Cx32ΔTg group

Next, we observed the effect of consumption of the HFD with DMN injections at 17 weeks. The body, liver, and fat weights; serum TP; albumin; and ALT levels were significantly elevated in the HFD groups for both genotypes (Tables 1, 2). Serum AST was significantly increased by the HFD only in Cx32ΔTg rats, and both AST and ALT were significantly higher in Cx32ΔTg as compared with Wt rats (Table 2; *P* < 0.01 and 0.05, respectively). As shown in Fig. 2a, the livers were enlarged and light-brown tinged with white in the HFD groups for both genotypes. There was diffuse lipid accumulation with the appearance of neutrophil clusters and hepatocellular ballooning in the Wt-HFD and Cx32ΔTg-HFD groups, and these changes in Cx32ΔTg rats were more pronounced (Fig. 2a–d). As determined from the sum of steatosis, lobular inflammation, and ballooning injury scores, NAS was elevated in the HFD groups, and the score was significantly higher in Cx32ΔTg than in Wt rats (Fig. 2e; *P* < 0.05). These results suggest that Cx32 dysfunction enhances NASH-related hepatotoxicity in the HFD–DMN model, as was the case with the Cx32ΔTg-MCDD model in our previous report (Sagawa et al. 2015). We further evaluated the insulin sensitivity in rats with HFD–DMN-induced NASH. A significant elevation of blood sugar and insulin levels by HFD feeding was observed only in Cx32ΔTg rats (Table 3; *P* < 0.05 and 0.001, respectively), resulting in a significantly higher HOMA-IR score in the Cx32ΔTg-HFD group (Table 3; *P* < 0.01 versus Tg-Ctrl, *P* < 0.05 versus Wt-HFD). Therefore, HFD and DMN stimulate fat accumulation and an inflammatory response in the liver and induce IR.

**Table 1:** Final body and organ weights in connexin 32 dominant-negative transgenic and wild-type rats treated with high-fat diet at week 17

	No. of rats	Body (g)	Liver		Kidney		Fat	
			Absolute (g)	Relative (%)	Absolute (g)	Relative (%)	Absolute (g)	Relative (%)
Wt-Control	10	516.5 ± 45.4	10.33 ± 1.38	1.99 ± 0.11	2.38 ± 0.19	0.46 ± 0.03	11.07 ± 3.10	2.12 ± 0.44
Wt-HFD	15	572.6 ± 39.5**	11.55 ± 1.18*	2.02 ± 0.12**	2.47 ± 0.16	0.43 ± 0.03	15.76 ± 3.30**	2.73 ± 0.43**
Cx32ΔTg − Control	10	526.6 ± 44.9	10.51 ± 1.40	1.99 ± 0.12	2.43 ± 0.20	0.46 ± 0.04	11.00 ± 3.83	2.05 ± 0.57
Cx32ΔTg-HFD	15	581.4 ± 54.2*	12.31 ± 1.69*	2.13 ± 0.21**	2.58 ± 0.16	0.45 ± 0.03	15.91 ± 3.13**	2.71 ± 0.36**

*Wt* wild-type, *HFD* high-fat diet, *Cx32ΔTg* connexin 32 dominant-negative transgenic

Tukey’s multiple comparison test *P < 0.05, **P < 0.01 vs genotype-matched control

**Table 2:** Serum levels of hepatic enzymes in connexin 32 dominant-negative transgenic and wild-type rats treated with high-fat diet at week 17

	No. of rats	TP (g/dl)	Alb (g/dl)	T-chol (mg/dl)	LDL (mg/dl)	HDL (mg/dl)	AST (U/l)	ALT (U/l)	ALP (U/l)
Wt-Control	10	6.3 ± 0.2	4.1 ± 0.2	61.8 ± 7.5	8.8 ± 1.4	32.4 ± 4.7	71.7 ± 15.1	33.3 ± 4.0	309.7 ± 65.0
Wt-HFD	15	6.0 ± 0.2**	3.9 ± 0.2**	62.2 ± 8.8	9.3 ± 2.0	35.5 ± 6.7	78.9 ± 13.7	52.0 ± 9.3***	357.0 ± 101.2
Cx32ΔTg − Control	10	6.2 ± 0.3	4.0 ± 0.2	64.5 ± 12.4	10.2 ± 2.9	32.9 ± 6.9	74.9 ± 13.8	35.5 ± 6.2	471.3 ± 403.8
Cx32ΔTg-HFD	15	5.9 ± 0.2**	3.7 ± 0.2**	66.3 ± 13.5	11.7 ± 3.6^#^	36.2 ± 7.7	102.5 ± 25.5**^, ##^	64.4 ± 15.0***^, #^	528.1 ± 140.4

*Wt* wild-type, *HFD* high-fat diet, *Cx32ΔTg* connexin 32 dominant-negative transgenic, *TP* total protein, *Alb* albumin, *T-chol* total cholesterol, *LDL* low-density lipoprotein cholesterol, *HDL* high-density lipoprotein cholesterol, *AST* aspartate aminotransferase, *ALT* alanine aminotransferase, *ALP* alkaline phosphatase

Tukey’s multiple comparison test *P < 0.05, **P < 0.01, ***P < 0.001 vs genotype-matched control, ^#^*P* < 0.05, ^##^*P* < 0.01 vs Wt-HFD

**Table 3:** Insulin resistance in connexin 32 dominant-negative transgenic and wild-type rats treated with high-fat diet at week 17

	No. of rats	BS (mg/dl)	Insulin (μU/ml)	HOMA-IR
Wt-Control	10	136.7 ± 21.4	21.4 ± 13.3	7.5 ± 5.1
Wt-HFD	15	159.3 ± 15.6	24.5 ± 9.6	9.8 ± 4.0
Cx32ΔTg − Control	10	137.0 ± 16.5	15.6 ± 4.6	5.4 ± 2.1
Cx32ΔTg-HFD	15	162.3 ± 24.8*	44.3 ± 25.8***^, #^	18.7 ± 13.0**^, #^

*Wt* wild-type, *HFD* high-fat diet, *Cx32ΔTg* connexin 32 dominant-negative transgenic, *HOMA-IR* homeostasis model assessment-insulin resistance

Tukey’s multiple comparison test **P < 0.01, ***P < 0.001 vs genotype-matched control, ^#^*P* < 0.05 vs Wt-HFD

### Combination of HFD with DMN induces fibrosis in Cx32ΔTg rats

**Fig. 3:**
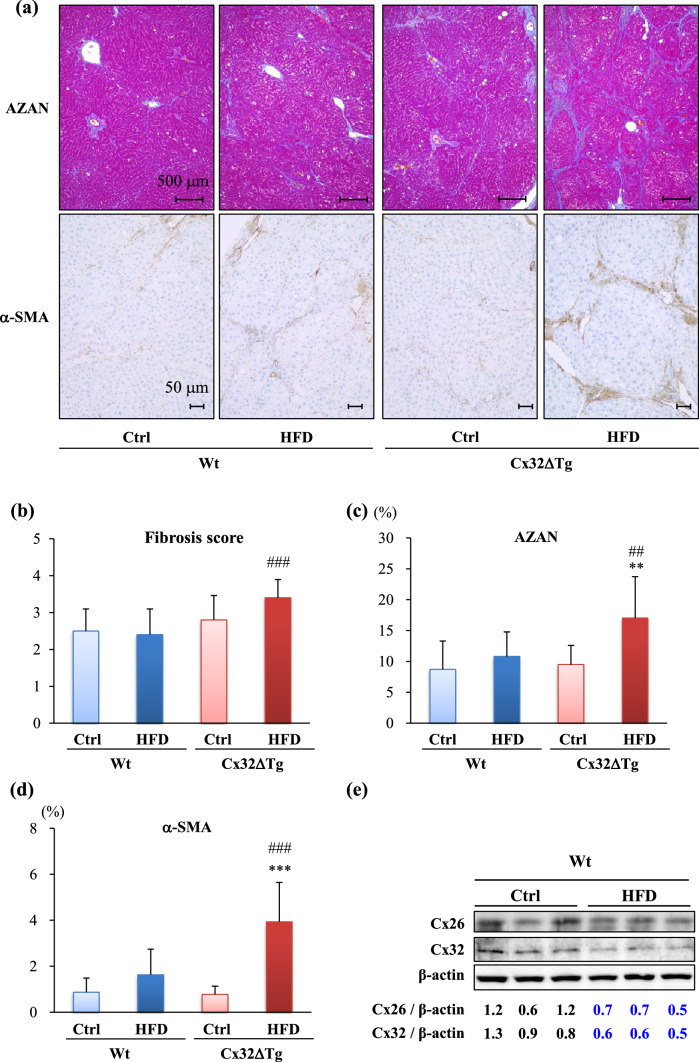
Fibrosis induced by high-fat diet (HFD) and dimethylnitrosamine (DMN) in Cx32 dominant-negative transgenic (Cx32ΔTg) rats. **a** Azan staining and immunohistochemistry for α-smooth muscle actin (α-SMA) in liver sections from Control (Ctrl) and HFD-treated wild-type (Wt) or Cx32ΔTg group at week 17. Fibrotic regions were stained blue by Azan staining. Fibrosis score (**b**) and percentage of fibrosis area (**c**) were evaluated by Azan staining. **d** Percentage of α-SMA-positive area. Data are presented as mean ± SD, *n* = 16–21 per group, **P <0.01, ***P <0.001 statistically significant between genotype-matched Ctrl and HFD groups, ^##^*P *<0.01, ^###^*P *<0.001 statistically significant between treatment-matched Wt and Cx32ΔTg group. **e** Western blotting for Cx32 and Cx26 protein in liver from each group. Each lane represents an individual rat

Liver fibrosis can occur subsequent to continuous chronic hepatitis, including in NASH. Thus, we evaluated fibrosis changes in Wt and Cx32ΔTg rats. HFD only did not induce fibrosis in both Wt and Cx32ΔTg rats at 5 weeks (Fig. S1a). Azan staining revealed that intake of an HFD plus DMN injection caused fibrosis mainly around the portal tracts, and partially in the central zones in Wt rats at 17 weeks (Fig, 3a). Furthermore, more progressive fibrosis with the extension of fibrous septa and bridging from the portal to the portal or centrilobular area was observed in Cx32ΔTg rats (Fig. 3a). The histological scores for fibrosis were elevated only in the Cx32ΔTg-HFD group, and it was significantly higher as compared with the Wt-HFD groups (Fig. 3b; *P* < 0.001). The percentage of the fibrotic area (stained blue by Azan) was significantly higher in the Cx32ΔTg-HFD group than in the Cx32ΔTg-Ctrl (Fig. 3c; *P* < 0.01) and Wt-HFD (*P* < 0.01) groups. Immunostaining of α-SMA showed increased myofibroblasts along with collagen accumulation in the Cx32ΔTg-HFD group (Fig. 3d; *P* < 0.001 versus Tg-Ctrl and Wt-HFD). We previously confirmed that Cx32 and Cx26 in hepatocytes were gradually reduced during the progression of MCDD-induced NASH, and the anti-oxidant luteolin attenuated the decreased expression and prevented steatohepatitis in Wt rats (Sagawa et al. 2015). In the present study, western blotting indicated that Cx32 and Cx26 protein levels were also decreased by HFD treatment in Wt rats (Fig. 3e). Together these results suggest that Cx32 and Cx26 may be necessary to protect the liver against NASH-related liver injury.

### Hepatocarcinogenesis during NASH is promoted in Cx32ΔTg–HFD–DMN model

**Fig. 4:**
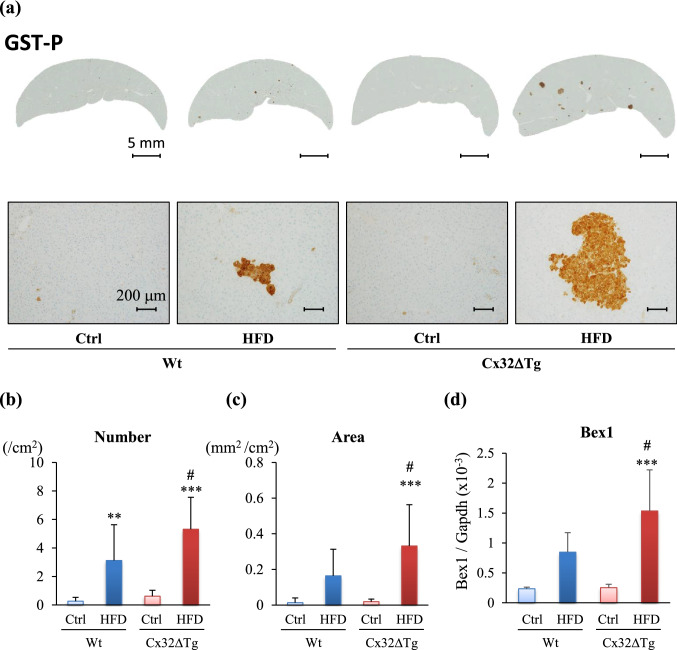
Hepatocarcinogenesis in nonalcoholic steatohepatitis (NASH) in Cx32 dominant-negative transgenic (Cx32ΔTg) rats receiving high-fat diet (HFD) and dimethylnitrosamine (DMN). **a** Representative foci positive for glutathione S-transferase placental form (GST-P) in liver sections from the Control (Ctrl), HFD-treated wild-type (Wt), and Cx32ΔTg groups at week 17. Number (**b**) and area (**c**) of GST-P-positive hepatic foci. Data are presented as mean ± SD, *n* = 16–21 per group, and (**d**) mRNA level of brain expressed, X-linked 1 (Bex1) was measured by quantitative RT-PCR. Data are presented as mean ± SD, *n* = 6 per group, **P <0.01, ***P <0.001 statistically significant between genotype-matched Ctrl and HFD groups, ^#^*P *<0.05 statistically significant between treatment-matched Wt and Cx32ΔTg groups

To explore the effect of HFD and DMN on hepatocarcinogenesis in Wt and Cx32ΔTg rats, the number and area of GST-P positive foci were quantitated (Fig. 4a–c). The area of GST-P positive foci was significantly expanded by HFD treatment only in Cx32ΔTg rats (*P* < 0.001 versus Tg-Ctrl, and *P* < 0.05 versus Wt-HFD), although the values were numerically increased by HFD in both Wt and Cx32ΔTg rats. We previously determined that *Bex1* is up-regulated in GST-P positive foci in Cx32ΔTg rats with MCDD-induced NASH. In this study, the Bex1 mRNA level in Cx32ΔTg-HFD groups was also higher than that in Cx32ΔTg-Cont (*P* < 0.001) and Wt-HFD (*P* < 0.05), and it was correlated with the area of GST-P positive foci (Fig. 4d). These results suggest that the combination of an HFD and DMN has the potential to initiate a carcinogenic response in hepatocytes and that Cx32 dysfunction promotes hepatocarcinogenesis in NASH.

### Inflammatory cytokines are induced with activation of NF-κB and SAPK/JNK signaling during NASH development in Cx32ΔTg rats

**Fig. 5:**
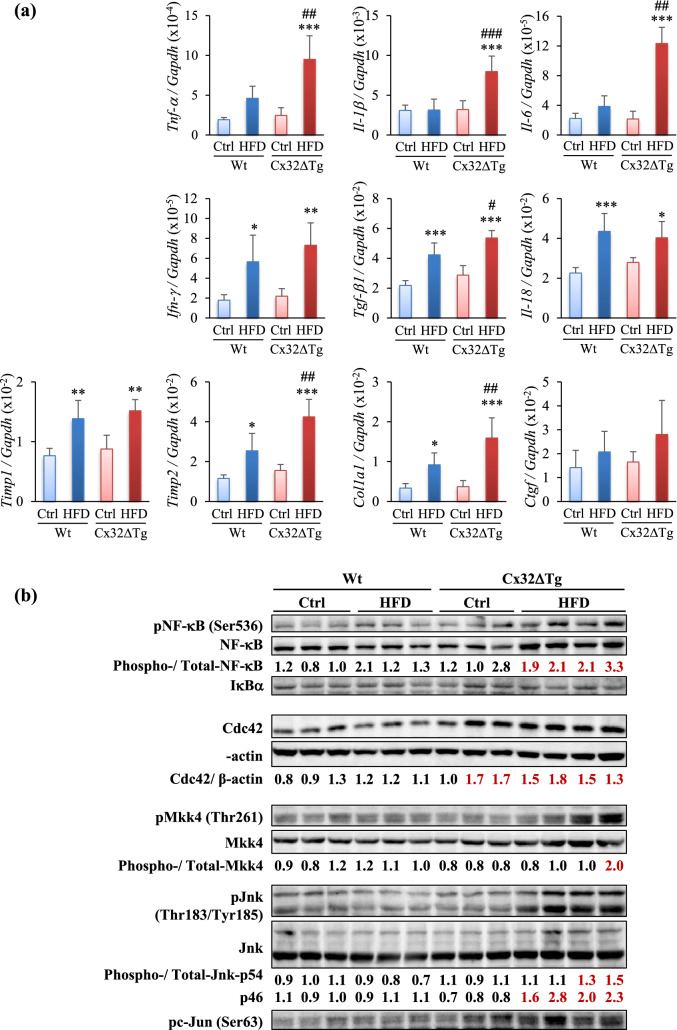
Up-regulation of inflammatory cytokines and activation of NF-κB and JNK signaling in NASH induced in Cx32 dominant-negative transgenic (Cx32ΔTg) rats. **a** mRNA level for inflammatory cytokines *Il-18, Tnf-α*, *Il-1β*, *Il-6*, *Ifn-γ*, *Tgf-β1*, *Il-18*, *Timp1*, *Timp2*, *Col1a1*, and *Ctgf* as measured by quantitative RT-PCR. Data are presented as mean ± SD, *n* = 6 per group, *P <0.05, **P <0.01, ***P <0.001 statistically significant between genotype-matched Ctrl and HFD groups, ^#^*P *<0.05, ^##^*P *<0.01, ^###^*P *<0.001 statistically significant between treatment-matched Wt and Cx32ΔTg group. **b** Protein levels of NF-κB signaling proteins (NF-κB, pNF-κB, IκB-α), SAPK/JNK signaling proteins (Cdc42, Mkk, pMkk, Jnk, pJnk, and pc-Jun) were measured by western blotting. Each lane represents an individual rat

A growing body of evidence supports that inflammatory cytokines such as TNF-α, TGF-β1, and IL-6, as well as inflammasome-related factors such as IL-1β, IL-18, play a key role in the progression of NASH (Dela Pena et al. 2005; Henao-Mejia et al. 2012; Seki et al. 2007; Wieckowska et al. 2008). Regarding liver fibrosis, we confirmed that not only *Tgf-β1* mRNA expression but also that of *Col1a1*, *Timp*s, and *Ctgf* mRNA expressions were correlated with liver fibrosis in the Cx32ΔTg-MCDD model (Sagawa et al. 2015). Therefore, we further investigated differences in the activity of inflammatory signaling between Wt and Cx32ΔTg rats by analyzing the expression level of these inflammatory cytokines. The mRNA expression of *Tnf*-*a*, *Il*-*1b*, *and Il*-*6* was significantly elevated by the HFD only in Cx32ΔTg rats, *Ifn*-*g*, *Tgf*-*b1*, *Il*-*18*, *Timp1*, *Timp2,* and *Col1a1* were significantly elevated by HFD consumption in both Wt and Cx32ΔTg rats. The level of these cytokines was higher in the Cx32ΔTg-HFD group than in the Wt-HFD group (Fig. 5a). A previous study indicated that the expression of NF-κB and Bex1 were elevated in NASH induced by MCDD in Cx32ΔTg rats. Furthermore, the up-regulation of Bex1 increased cell proliferation through the activation of NF-κB and JNK/SAPK signaling in HCC cells (Sagawa et al. 2015). Therefore, we analyzed whether NF-κB and JNK/SAPK signaling is activated in rats with NASH induced by an HFD in combination with DMN. Western blotting showed that the HFD led to an increase in pNF-κB in the Cx32ΔTg-HFD group (Fig. 5b). In accordance with JNK/SAPK signaling, protein expression of Cdc42, pMkk4, pJnk, and pc-Jun were increased in the Cx32ΔTg-HFD group. These results suggest that NF-κB and JNK/SAPK signaling are activated and involved in the progression of inflammation and carcinogenesis in Cx32ΔTg rats in the HFD–DMN NASH model.

## Discussion

NAFLD is a chronic liver disease characterized by fat deposition in the liver, and is associated with obesity, hyperlipidemia, arterial hypertension, and IR in type 2 diabetes (Marchesini et al. 2003; Rinella 2015). These factors related to metabolic syndrome can promote progression of NAFLD to NASH, which is defined not only by the presence of steatosis but also lobular inflammation and hepatocyte ballooning with or without fibrosis (Kleiner et al. 2005; Ludwig et al. 1980; Yu et al. 2016). Therefore, an appropriate animal model for NAFLD/NASH is required to reflect not only liver histopathology but also the pathophysiology of metabolic syndrome. However, multiple stages being involved in the progression in NAFLD/NASH, and that it is difficult to reproduce all of these stages in a single animal model.

HFD feeding, which is one of the most popular methods for reproducing NAFLD in animal models, induces steatosis in the liver, obesity and IR (Asgharpour et al. 2016; Santhekadur et al. 2018). A recent study reported that intake of an HFD with 5% cholesterol and 40% fat content, i.e., a steatohepatitis-inducing HFD (STHD-01), resulted in bridging fibrosis and tumor formation at around 36 weeks, in addition to induction of steatosis within only 1 week (Ejima et al. 2016). These studies suggest that hepatic pathological changes induced by HFD are not severe, which limits the use of this model for the examination of NAFLD or early stages of NASH. On the other hand, the MCDD model exhibits similar hepatic histological changes to human NASH; MCDD model has the advantage of inducing steatohepatitis in a shorter period of time (less than 10 weeks) than the HFD model, as well as inducing fibrosis, increased pro-inflammatory cytokine levels, and oxidative stress (Tanaka et al. 2012; Yamaguchi et al. 2008). Furthermore, our previous study indicated that bridging fibrosis or cirrhosis can be induced by 12-week MCDD treatment in Cx32ΔTg rats, and those effects were attenuated by luteolin, which acts as an anti-oxidant in vivo (Iida et al. 2020; Naiki-Ito et al. 2019; Sagawa et al. 2015). However, the MCDD model does not replicate other metabolic features of NASH, including obesity, IR, or hyperlipidemia due to modulation of sugar and lipid metabolism via choline deficiency (Machado et al. 2015). In the present study, HFD and DMN led to not only steatohepatitis and fibrosis but also preneoplastic hepatic lesions in Cx32ΔTg rats. Furthermore, elevation levels of the inflammatory cytokine, NF-κB, as well as JNK signaling and IR occurred in the present model. Therefore, the Cx32ΔTg–HFD–DMN model may be useful both for screening drug efficacy against hepatotoxicity as well as parameters related to metabolism in NASH.

Imbalance of fatty acid synthesis and lipolysis causes fat accumulation in the adipose tissue and the liver, resulting in IR, which represents a failure of insulin to transport glucose to the target cells and consequent reduction of blood sugar levels (Donnelly et al. 2005). However, IR can induce lipolysis and the release of free fatty acids from the adipose tissue, leading to elevated levels of circulating fatty acids and progression from simple steatosis to steatohepatitis (Eguchi et al. 2006). Thus, the development of IR and NAFLD is thought to be closely linked. In the present study, body, liver, and visceral fat weights, as well as liver steatosis, were increased by HFD feeding in both Wt and Cx32ΔTg rats, and there was no significance in effect between the genotypes. On the other hand, the degree of hepatocyte injury and lobular inflammation was more severe in Cx32ΔTg rats as compared to Wt rats. Moreover, the elevation of the HOMA-IR score due to increased blood sugar and plasma insulin was only observed in the Cx32ΔTg-HFD groups. This is a novel finding, demonstrating that dysfunction of hepatic Cx32 affects not only the liver but also the entire body; NASH progression induced by Cx32 dysfunction exacerbates IR and subsequently triggers a vicious cycle within the liver and throughout the body. Further research on transcriptomic and proteomic analysis may help to further understand the global effect of Cx32 in the NASH model.

Liver fibrosis is a pathological reaction that occurs as a result of various types of chronic liver disease and characterized by the accumulation of abundant extracellular matrix, and it destroys the physiological architecture of the liver as a result of irreversible remodeling (Trautwein et al. 2015; Tsuchida and Friedman 2017). Hepatic stellate cells (HSCs) are responsible for initiating fibrogenesis (Tsuchida and Friedman 2017). In liver injury, HSCs are activated from a quiescent to a myofibroblast-like active phenotype by damaged hepatocytes and Kupffer cells, which results in their expression of α-SMA and production of collagen (Wang et al. 2010). HSC activation is mediated by inflammatory cytokines such as TGF-β1 and TNF-α (Okina et al. 2020; Seki et al. 2007). TGF-β1 is the most potent mediator activated by NF-κB (Seki et al. 2007); however, some studies have indicated that TGF-β1 can trigger fibrogenesis through activation of NF-κB (Liu et al. 2015). In the present study, NF-κB activation and up-regulation of TGF-β1 and TNF-α were triggered as a result of NASH induced by an HFD combined with DMN injections in Cx32ΔTg rats, even though these changes were smaller in the liver of Wt rats. Notably, liver fibrosis was significantly more severe in Cx32ΔTg rats that received HFD and DMN. These results suggest that NF-κB and TGF-β1 play crucial roles in hepatic fibrogenesis.

In summary, the combination of HFD and DMN-induced steatohepatitis and fibrosis with the enhancement of inflammatory cytokines and activation of NF-κB and JNK signaling in the liver of Cx32ΔTg rats; as well as hepatic histological changes, obesity, and IR were observed. These pathophysiological findings in this model closely resemble the features of NASH in human. Moreover, hepatocarcinogenesis was significantly increased by the HFD with DMN injection in Cx32ΔTg rats. These results indicate that Cx32ΔTg–HFD–DMN NASH model is useful for exploring the mechanisms of NASH and for screening the efficacy of potential pharmacological treatments for NASH.

## Electronic supplementary material

Below is the link to the electronic supplementary material.

Supplementary material 1 (PDF 479 kb)

## Abbreviations

ALT: Alanine aminotransferase
AST: Aspartate aminotransferase
Bex1: Brain expressed, X-linked 1
Ctrl: Control
Cx: Connexin
Cx32ΔTg: Cx32 dominant-negative transgenic
DMN: Dimethylnitrosamine
GST-P: Glutathione *S*-transferase placental form
HCC: Hepatocellular carcinoma
HFD: High-fat diet
HOMA-IR: Homeostasis model assessment-insulin resistance
HSC: Hepatic stellate cells
IR: Insulin resistance
MCDD: Methionine choline-deficient diet
NAFLD: Non-alcoholic fatty liver disease
NAS: Non-alcoholic fatty liver disease activity score
NASH: Non-alcoholic steatohepatitis
NF-κB: Nuclear factor-κB
ROS: Reactive oxygen species
α-SMA: α-smooth muscle actin
Wt: Wild-type
