# Supplementary material for: A novel model of non-alcoholic steatohepatitis with fibrosis and carcinogenesis in connexin 32 dominant-negative transgenic rats
Source: Arch Toxicol. 2020 Aug 24;94(12):4085–97. doi: 10.1007/s00204-020-02873-5 (PMC7655588; doi:10.1007/s00204-020-02873-5)
Supplement: Supplementary file 1 — Supplementary material 1 (PDF 479 kb) [file 204_2020_2873_MOESM1_ESM.pdf]

Table S1. Sequence of primers used for quantitative RT-PCR.

Gene	Forward	Reverse
Bex1	CAGGTCTGAGGACCAGAAGC	CAACCGGCTCCCTTTTGATG
Tnf-α	ACTGAACTTCGGGGTGATCG	GCTTGGTGGTTTGCTACGAC
Il-1β	AGCTGAAAGCTCTCCACCTC	GTGCCGTCTTTCATCACACAG
Il-6	TCCGGAGAGGAGACTTCACA	ACAGTGCATCATCGCTGTTC
Ifn-γ	GCATTCATGAGCATCGCCAA	AGATTCTGGTGACAGCTGGTG
Tgf-β1	GTCAACTGTGGAGCAACACG	TTCCGTCTCCTTGGTTCAGC
Il-18	TGACAAAAGAAACCCGCCTG	TCACAGATAGGGTCACAGCC
Timp1	GTAAAGCCTGTAGCTGTGCC	CCACAGCGTCGAATCCTTTG
Timp2	CCAAAGCAGTGAGCGAGAAG	TCGATGTCCTTGTCAGGTCC
Col1a1	TGACGCATGGCCAAGAAGA	CGTGCCATTGTGGCAGATAC
Ctgf	CAGGCTGGAGAAGCAGAGTC	CCGGTAGGTCTTCACACTGG
Gapdh	GCATCCTGCACACCAACTG	GCCTGCTTCACCACCTTGTT

Table S2. Final body and organ weights in connexin 32 dominant negative transgenic and wild-type rats treated with high fat diet at week 5.

	No. of rats	Body (g)	Liver		Kidney		Fat	
			Absolute (g)	Relative (%)	Absolute (g)	Relative (%)	Absolute (g)	Relative (%)
Wt-Control	6	434.3 ± 13.8	10.73 ± 0.68	2.47 ± 0.16	2.30 ± 0.13	0.53 ± 0.03	7.81 ± 1.33	1.79 ± 0.26
Wt-HFD	6	465.4 ± 21.0*	12.62 ± 0.61***	2.71 ± 0.09***	2.37 ± 0.14	0.51 ± 0.02	11.17 ± 0.68***	2.41 ± 0.23**
Cx32ΔTg-Control	6	417.3 ± 34.8	10.16 ± 1.28	2.43 ± 0.12	2.27 ± 0.21	0.54 ± 0.03	6.97 ± 2.12	1.65 ± 0.40
Cx32ΔTg-HFD	6	461.0 ± 11.5*	12.66 ± 0.91**	2.75 ± 0.18**	2.33 ± 0.15	0.51 ± 0.03	10.82 ± 2.64*	2.34 ± 0.56*

Wt, wild-type; HFD, high fat diet; Cx32ΔTg, connexin 32 dominant negative transgenic

Tukey's multiple comparisons test *: P<0.05, **: P<0.01, ***: P<0.001 vs genotype-matched control

Table S3. Serum levels of hepatic enzymes in connexin 32 dominant negative transgenic and wild-type rats treated with high fat diet at week 5.

	**No. of rats**	**TP (g/dl)**	**Alb (g/dl)**	**T-chol (mg/dl)**	**LDL (mg/dl)**	**HDL (mg/dl)**	**AST (U/l)**	**ALT (U/l)**	**ALP (U/l)**
Wt-Control	6	6.3 ± 0.2	4.0 ± 0.1	60.0 ± 10.7	6.6 ± 1.5	34.7 ± 6.0	68.8 ± 7.2	34.0 ± 5.1	518.5 ± 52.2
Wt-HFD	6	6.2 ± 0.1	4.0 ± 0.1	65.2 ± 17.1	8.5 ± 3.3	39.5 ± 11.7	74.8 ± 13.2	55.7 ± 8.1***	606.2 ± 99.0
Cx32ΔTg-Control	6	6.3 ± 0.1	4.1 ± 0.1	58.5 ± 13.8	7.8 ± 2.0	35.7 ± 9.3	72.5 ± 13.0	36.2 ± 4.9	860.8 ± 297.0
Cx32ΔTg-HFD	6	6.2 ± 0.2	4.0 ± 0.1	65.8 ± 8.4	9.2 ± 1.1	39.2 ± 6.2	87.7 ± 15.2	62.2 ± 9.3***	748.8 ± 229.4

Wt, wild-type; HFD, high fat diet; Cx32ΔTg, connexin 32 dominant negative transgenic; TP, total protein; Alb, albumin; LDL, low-density lipoprotein cholesterol; HDL, high-density lipoprotein cholesterol; AST, aspartate aminotransferase; ALT, alanine aminotransferase; ALP, alkaline phosphatase

Tukey's multiple comparisons test ***: P<0.001 vs genotype-matched control

(a)

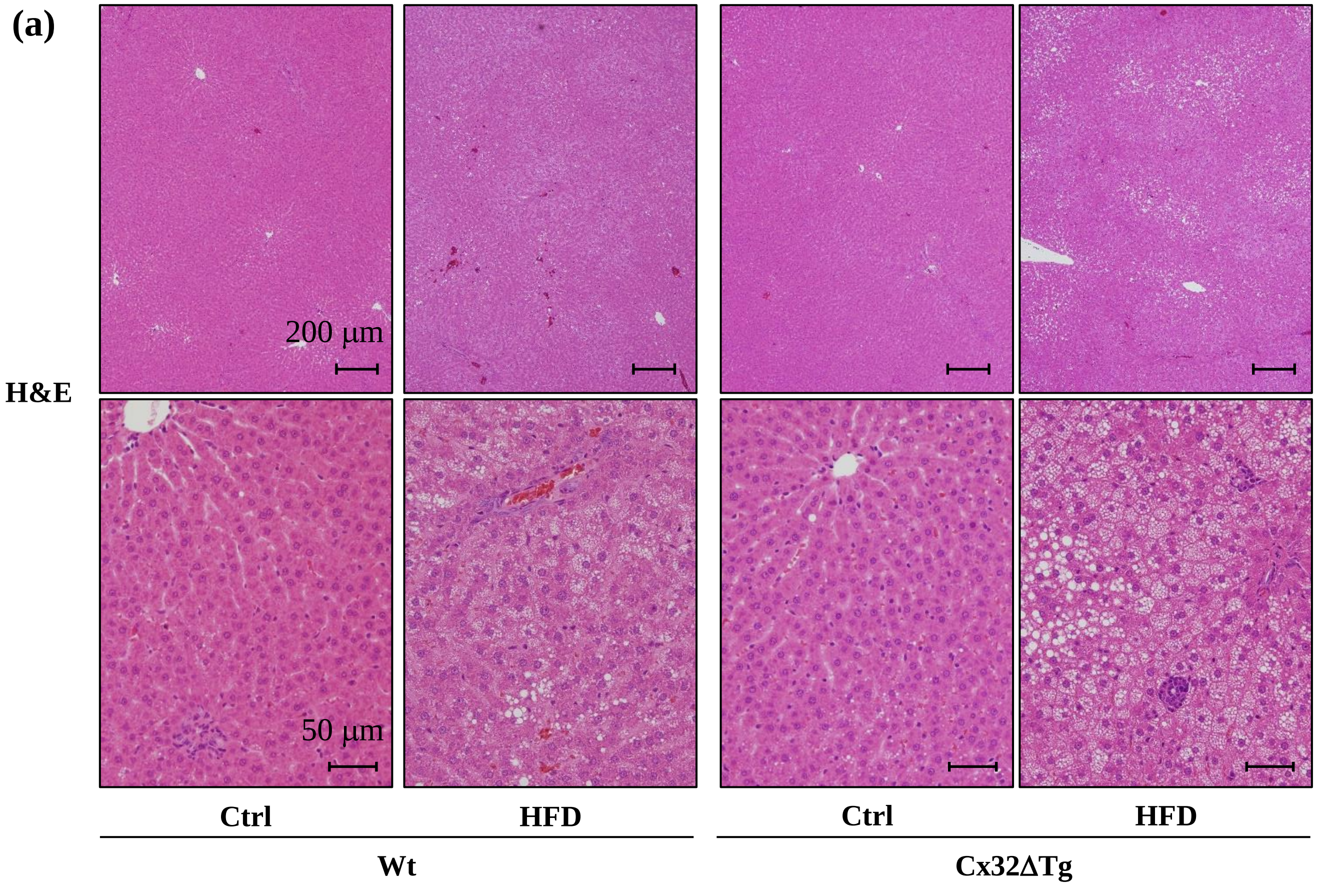

(b)

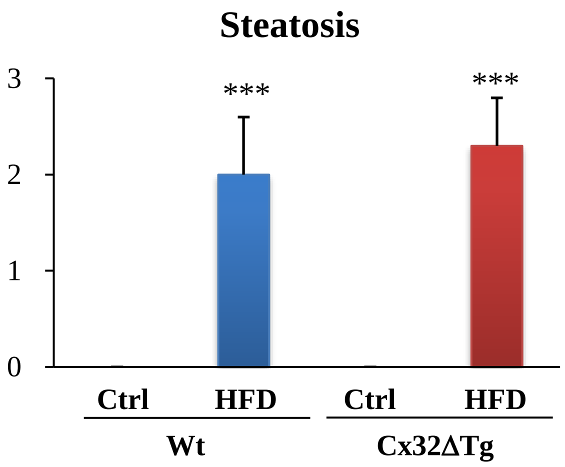

(c)

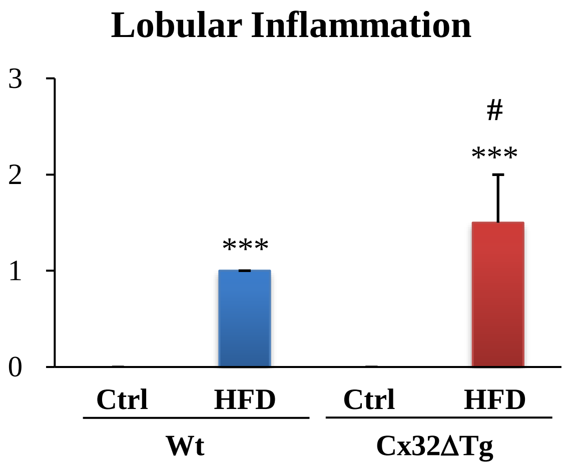

(d)

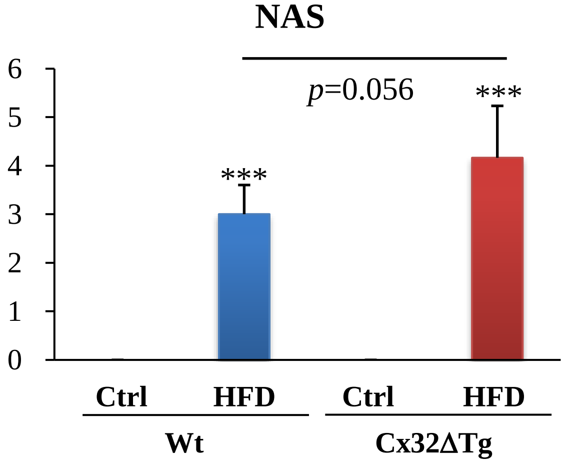

Fig. S1. Steatohepatitis induced by high fat diet (HFD) in Cx32 dominant negative transgenic (Cx32ΔTg) rats at week 5. (A) Representative macroscopic pictures and hematoxylin and eosin (H&E) staining of liver sections from the Control (Ctrl), HFD-treated wild-type (Wt), and Cx32ΔTg groups at week 5 prior to administration of dimethylnitrosamine. (b-d) Histopathological analysis of steatohepatitis with severity scores including steatosis (b), lobular inflammation (c), and non-alcoholic fatty liver disease activity score (NAS) (d). Data are presented as mean ± SD, n = 6 per group, ****P* < 0.001 statistically significant between genotype-matched Ctrl and HFD groups, #*P* < 0.05 statistically significant treatment-matched Wt and Cx32ΔTg group.
